# The Proteoglycan Glycomatrix: A Sugar Microenvironment Essential for Complement Regulation

**DOI:** 10.3389/fimmu.2013.00412

**Published:** 2013-11-26

**Authors:** Simon J. Clark, Paul N. Bishop, Anthony J. Day

**Affiliations:** ^1^Centre for Hearing and Vision Research, Institute of Human Development, Faculty of Medicine and Human Sciences, University of Manchester, Manchester, UK; ^2^Wellcome Trust Centre for Cell Matrix Research, Faculty of Life Sciences, University of Manchester, Manchester, UK

**Keywords:** proteoglycans, glycosaminoglycans, glycomatrix, complement regulation, tissue specificity

Proteoglycans (PGs) are major components of all mammalian tissues, being present ubiquitously on cell surfaces and within extracellular matrices (ECM). They play vital roles in mammalian physiology and have been implicated in many disease processes. PGs comprise a single core protein with one or more glycosaminoglycan (GAG) chains attached where these un-branched polysaccharides are composed of repeating disaccharide units that show considerable diversity in their disaccharide composition, glycosidic linkages, and levels/positions of sulfation (1, 2). GAGs, therefore, contain huge numbers of structural permutations (even in the same chain), representing a vast possible array of diverse structures that can determine the fate of local environments (3): i.e., through their modulation of protein-binding and activity. Thus, PGs constitute a tissue and region-specific microenvironment of sugar molecules, both within the ECM and at the cell-matrix interface, which acts as a local regulator of tissue function and homeostasis. As described below, it is our opinion that this proteoglycan “glycomatrix” plays a key role in the regulation of the immune system by acting as a molecular postcode that controls local immune function (4). Here we will illustrate this with examples of the effects of PGs/GAGs on the immune system in the eye, heart, kidney, and lung. In particular, we will focus on recent evidence that GAGs can positively and negatively regulate the alternative pathway of complement and suggest how the dysregulation of this aspect of innate immunity may contribute to disease processes in a tissue-specific manner.

## Proteoglycans Provide a Diverse Molecular Postcode for Protein Regulation

Despite the diversity possible in GAG sequences, considerable specificity in chain composition is seen between and within different tissues (5, 6). For example, in the human eye it has been shown that specific GAG structures and PG core proteins are located in defined layers of the retina, resulting in remarkable compartmentalization even within the same organ (6, 7); this has the potential to regulate the binding/function of proteins, such as those that control angiogenesis and innate immunity. Importantly, the glycomatrix can provide a postcode that can be distinguished by different proteins [*via* their abilities to recognize distinct GAG structures (4)], whereby even members of the same protein family [e.g., the interleukin cytokines (8)], can bind differentially, i.e., at “defined” sites.

At present, because of the current limitations in GAG sequencing, there is relatively little detailed information available on the precise structures of glycomatrix postcodes found in different tissues or their protein-binding specificities. What is clear, however, is that GAGs/PGs play an important role in the recruitment and regulation of a wide range of proteins, including modulators of the innate/cellular immune system, as well as those that are involved in tissue remodeling during inflammatory/disease processes. For instance, GAGs have been found to be key elements in regulating pulmonary inflammation during lung infection through their binding of cytokines, chemokines, and growth factors, which promotes leukocyte adhesion and accumulation (9). The binding of cytokines and chemokines to PGs conceals proteolytic cleavage sites: for example, heparan sulfate (HS) limits the proteolytic digestion of interferon-gamma, which increases its activity sixfold (10). Furthermore, GAGs specifically bind matrix metalloproteinases (MMPs) as well as their endogenous inhibitors, the tissue inhibitors of metalloproteinases (TIMPs). This means that PGs are able to sequester MMPs and TIMPs in specific regions of the lung leading to direct regulation of enzyme activity; e.g., following acute lung injury or infection (9). Other examples include chondroitin 4-sulfate playing a direct role in the presentation of pro-MMP2 to MT3-MMP (where chondroitin 6-sulfate or HS do not do this), thus leading to its activation (11), while on the other hand HS can recruit and inhibit ADAM12 (12).

## Modulation of the Complement System by Proteoglycans

The PG glycomatrix can influence the innate immune system *via* recruitment of regulatory factors from the blood; e.g., the positive and negative regulators of the complement system, proderdin, and complement factor H (CFH). Properdin stabilizes the alternative pathway C3 convertase, promoting amplification of the complement cascade, leading to C3b deposition that labels targets for destruction by phagocytosis and allows formation of the membrane attack complex, which can lyse cells; this also leads to the production of pro-inflammatory mediators that attract leukocytes and cause mast cell degranulation. Conversely CFH, once recruited to a surface, can accelerate the decay of the C3 convertase and act as a co-factor for the proteolytic deactivation of C3b, thus dampening-down a complement response. This fine balance between positive and negative regulation can be greatly influenced by the composition of a tissue’s glycomatrix (13). While both properdin and CFH bind HS on renal tubular epithelial cells, they recognize distinct, non-overlapping, sequences within HS GAG chains; i.e., they do not compete for binding sites. It was reported that CFH only recognizes highly sulfated HS, while properdin is able to bind more lowly sulfated HS structures (e.g., those lacking N-sulfation) (14). Thus, this differential recognition of the glycomatrix likely allows both positive and negative regulators of the complement alternative pathway to be present together on the surface of these kidney cells, thus ensuring innate immune homeostasis (13). If this balance breaks down (e.g., due to impairment of protein/GAG interactions) it could lead to kidney damage and may explain the worsening of outcome in proteinuric patients, i.e., due to inappropriate complement activation.

The CFH protein is comprised of 20 complement control protein (CCP) domains where CCPs6–8 and CCPs19–20 mediate GAG-binding [see Ref. (15–17)]. Interestingly, our recent work has provided strong evidence that the HSPGs in the glomerular basement membrane (GBM) of the human kidney recruit CFH solely *via* its CCP19–20 surface recognition domain; this region of CFH recognizes highly sulfated HS structures (17). On the other hand, CCP6s–8 are largely responsible for CFH-binding to sites in the human eye, i.e., the retinal pigment epithelium (RPE) and the underlining Bruch’s membrane, a multi-layered ECM. We believe that this is because the GAG-binding specificities of the CCP6–8 and CCP19–20 regions are distinct (15, 17) and can therefore provide tissue specificity through recognition of different GAG structures (postcodes) in different tissue locations (see Table 1); i.e., they can distinguish different glycomatrices.

**Table 1:** **Comparison of the binding properties of the two GAG-binding regions of CFH**.

	CCP6–8		CCP19–20
	402Y	402H	
GAG CHAIN RECOGNITION^a^			
Hyaluronan	×	×	×
Dermatan sulfate	✓	✓	×
Chondroitin 4-sulfate	×	×	×
Chondroitin 6-sulfate	×	×	×
Heparan sulfate	✓	✓	✓
Heparin	✓	✓	✓
HEPARIN SULFATION SPECIFICITY^b^			
2-O desulfated	↓	↓ ↓	↓ ↓
6-O desulfated	↓	↓	↓ ↓
N-O desulfated	↓	↓ ↓ ↓	↓ ↓ ↓ ↓
TISSUE SPECIFICITY^c^			
Bruch’s membrane	+++	+	+
	(Broad specificity)	(Requires 2- and 6-O sulfation)	(Unknown)
RPE	+++	+++	+++
	(Broad specificity)	(Requires 2- and 6-O sulfation)	(Unknown)
Kidney GBM	−	−	+++

*^a^Based on direct binding experiments where ✓ means binding and × no binding*.

*^b^Binding to selectively desulfated preparations of heparin where ↓ means small reduction in binding, ↓ ↓ moderate reduction in binding, ↓ ↓ ↓large reduction in binding, and ↓ ↓ ↓ ↓ means no detectable binding*.

*^c^Based on the binding of fluorescently labeled protein (CCP6–8 and CCP19–20) probes to human tissue where − means no binding, + weak binding, ++ moderate binding, and +++ strong binding*.

Bruch’s membrane separates the RPE and photoreceptor cells in the neurosensory retina from the choroid, a vascular bed posterior to these structures. CFH, being the only secreted regulator of the alternative pathway, is solely responsible for protecting ECM such as Bruch’s membrane from complement-mediated damage (i.e., preventing complement amplification in healthy host tissues). We have found previously that CFH-binding sites in Bruch’s membrane are comprised mainly of HS, but with dermatan sulfate also playing a minor role (16). Moreover, we discovered that the Y402H polymorphism in the *CFH* gene [that changes a tyrosine to histidine in CCP7 (18)] impairs the ability of CFH to bind to GAG postcodes in Bruch’s membrane (16). This is likely to be important since this common polymorphism is strongly associated with the development of Age-related Macular Degeneration (AMD) (19–21), which is the most common form of blindness in the western world; individuals homozygous for the 402H form of CFH have a ~5-fold increased risk of developing AMD (20). Our studies have demonstrated that the disease-associated 402H variant has a rather restricted specificity, requiring highly sulfated structures, as opposed to the 402Y form which is able to bind a broader range of GAG sequences (15, 22). In the glycomatrix of the Bruch’s membrane the binding sites for the 402H variant of CFH are rare relative to those for 402Y (16). On this basis we hypothesize that insufficient binding of 402H within the Bruch’s membrane will lead to complement over-activation and local chronic inflammation, and thereby damage the RPE, contributing to the formation of the particulate deposits, called drusen, that are the hallmarks of AMD (16, 23, 24).

As noted above, the two GAG-binding regions in CFH have different specificities where these are likely to differentially regulate the interactions of this protein with sites in the eye and kidney (17). This may explain why mutations/polymorphisms within CCPs19–20 are associated with the kidney disease, atypical Hemolytic-Uremic Syndrome (aHUS), where uncontrolled complement activation is believed to lead to inflammation and the formation of blood clots, whereas, the Y402H polymorphism is linked to AMD. Patients suffering from aHUS do not present with any ocular phenotype and similarly AMD patients rarely have associated kidney problems. This provides a striking example of the tissue-specific nature of the glycomatrix microenvironment (e.g., of the Bruch’s membrane and GBM) and how this might differentially influence disease processes.

## Age-Related Effects on the Glycomatrix Postcode?

Alterations in the biosynthesis and turnover of PGs are known to occur with age (2), therefore concomitant effects on protein recruitment and tissue function would not be surprising. For example, there is an age-related change in the fine structure of HS that affects the migration of endothelial progenitor cells (25). Here the loss of a specific tri-sulfated disaccharide from their surface HS correlates with a reduction in their migratory response to vascular endothelial growth factor; this impairs the engraftment capacity of these cells, contributing to endothelial dysfunction and age-related vascular pathology. Similarly, human aorta HS is subject to age-related increases in the level of 6-O sulfation (26); this, in turn, leads to increased binding of platelet-derived growth factor resulting in its extracellular accumulation, which is hypothesized to facilitate aberrant smooth muscle cell migration and growth, i.e., in individuals prone to developing atherosclerotic disease. Another example is the recent finding that chondroitin sulfate and keratan sulfate chains of aggrecan, a major PG component of articular cartilage, decrease in both number and length with age, affecting amongst other things the mechanical properties of this tissue (27). Given the importance of GAGs in the regulation of complement (as described above), it is plausible that age-related changes in the glycomatrix of the eye could contribute to AMD pathogenesis, such that this might explain the age-related nature of this disease (15, 24).

## Proteoglycans as Targets for Therapeutics?

Given the major role played by PGs and their GAG chains in immune homeostasis, it seems plausible that they might make good therapeutic targets for immunological diseases. However, based on the above information, it will perhaps be prudent to attempt to modulate GAG-protein interactions in a tissue-dependent context. In this regard, it has recently been demonstrated that specific heparinoids, such as *N*- and *O*-sulfated K5 polysaccharides, can inhibit the binding of properdin to HS on renal tubular epithelial cells without affecting CFH, thereby controlling complement activation (13); this has the potential to prevent complement-derived tubular injury in proteinuric kidney diseases. Approaches of this type may be able to selectively inhibit the binding of pro-inflammatory proteins to particular GAG structures in a tissue/organ-specific manner and thus correct immune dysregulation in a wide range of pathological conditions.

## Concluding Remarks

In our opinion, the glycomatrix created by PGs remains an under-appreciated contributor to immune regulation. However, an increasing body of evidence is providing insights into just how important these complicated glycoproteins are in providing the fine control to immunological processes in tissue microenvironments, particularly within the ECM. More work is now needed to fully elucidate the biochemical basis of protein/GAG interactions and determine their roles in pathological processes. Further advances in our knowledge of the proteoglycan glycomatrix should facilitate the development of novel, tissue-specific, therapeutics, e.g., for diseases of the immune system.

## References

[1] Sarrazin S Lamanna WC Esko JD Heparan sulfate proteoglycans. Cold Spring Harb Perspect Biol (2011) 3:a004952 10.1101/cshperspect.a004952 21690215 PMC3119907

[2] Taylor KR Gallo RL Glycosaminoglycans and their proteoglycans: host-associated molecular patterns for initiation and modulation of inflammation. FASEB J (2006) 20:9–22 10.1096/fj.05-4682rev 16394262

[3] Schaefer L Schaefer RM Proteoglycans: from structural compounds to signaling molecules. Cell Tissue Res (2010) 339:237–46 10.1007/s00441-009-0821-y 19513755

[4] Langford-Smith A Keenan TDL Clark SJ Bishop PN Day AJ The role of complement in age-related macular degeneration: heparan sulphate, a ZIP code for complement factor H? J Innate Immun (2013) (in press). 10.1159/000356513 PMC4086042 24335201

[5] Lindahl B Eriksson L Lindahl U Structure of heparan sulphate from human brain, with special regard to Alzheimer’s disease. Biochem J (1995) 306:177–84 7864807 10.1042/bj3060177 PMC1136498

[6] Clark SJ Keenan TD Fielder HL Collinson LJ Holley RJ Merry CL Mapping the differential distribution of glycosaminoglycans in the adult human retina, choroid, and sclera. Invest Ophthalmol Vis Sci (2011) 52:6511–21 10.1167/iovs.11-7909 21746802 PMC3175996

[7] Keenan TD Clark SJ Unwin RD Ridge LA Day AJ Bishop PN Mapping the differential distribution of proteoglycan core proteins in the adult human retina, choroid, and sclera. Invest Ophthalmol Vis Sci (2012) 53:7528–38 10.1167/iovs.12-10797 23074202 PMC3493184

[8] Coombe DR Biological implications of glycosaminoglycan interactions with haemopoietic cytokines. Immunol Cell Biol (2008) 86:598–607 10.1038/icb.2008.49 18626488

[9] Gill S Wight TN Frevert CW Proteoglycans: key regulators of pulmonary inflammation and the innate immune response to lung infection. Anat Rec (2010) 293:968–81 10.1002/ar.21094 20503391 PMC4121077

[10] Lortat-Jacob H Baltzer F Grimaud JA Heparin decreases the blood clearance of interferon-gamma and increases its activity by limiting the processing of its carboxy-terminal sequence. J Biol Chem (1996) 271:16139–43 10.1074/jbc.271.27.16139 8663206

[11] Iida J Wilhelmson KL Ng J Lee P Morrison C Tam E Cell surface chondroitin sulfate glycosaminoglycan in melanoma: role in the activation of pro-MMP2 (pro-gelatinase A). Biochem J (2007) 403:553–63 10.1042/BJ20061176 17217338 PMC1876388

[12] Sørensen HP Vivès RR Manetopoulos C Albrechtsen R Lydolph MC Jacobsen J Heparan sulfate regulates ADAM12 through molecular switch mechanism. J Biol Chem (2008) 283:31920–32 10.1074/jbc.M804113200 18801731

[13] Zaferani A Vivès RR van der Pol P Navis GJ Daha MR van Kooten C Factor H and properdin recognize different epitopes on renal tubular epithelial heparan sulfate. J Biol Chem (2012) 287:31471–81 10.1074/jbc.M112.380386 22815489 PMC3438980

[14] Zaferani A Vivès RR van der Pol P Hakvoort JJ Navis GJ van Goor H Identification of tubular heparan sulfate as a docking platform for the alternative complement component properdin in proteinuric renal disease. J Biol Chem (2011) 286:5359–67 10.1074/jbc.M110.167825 21135110 PMC3037648

[15] Clark SJ Higman VA Mulloy B Perkins SJ Lea SM Sim RB His-384 allotypic variant of factor H associated with age-related macular degeneration has different heparin binding properties from the non-disease-associated form. J Biol Chem (2006) 281:24713–20 10.1074/jbc.M605083200 16787919

[16] Clark SJ Perveen R Hakobyan S Morgan BP Sim RB Bishop PN Impaired binding of the age-related macular degeneration-associated complement factor H 402H allotype to Bruch’s membrane in human retina. J Biol Chem (2010) 285:30192–202 10.1074/jbc.M110.103986 20660596 PMC2943316

[17] Clark SJ Ridge LA Herbert AP Hakobyan S Mulloy B Lennon R Tissue-specific host recognition by complement factor H is mediated by differential activities of its glycosaminoglycan-binding regions. J Immunol (2013) 190:2049–57 10.4049/jimmunol.1201751 23365078 PMC3672945

[18] Day AJ Willis AC Ripoche J Sim RB Sequence polymorphism of human complement factor H. Immunogenetics (1988) 27:211–4 10.1007/BF00346588 2962936

[19] Hageman GS Anderson DH Johnson LV Hancox LS Taiber AJ Hardisty LI A common haplotype in the complement regulatory gene factor H (HF1/CFH) predisposes individuals to age-related macular degeneration. Proc Natl Acad Sci U S A (2005) 102:7227–32 10.1073/pnas.0501536102 15870199 PMC1088171

[20] Sofat R Casas JP Webster AR Bird AC Mann SS Yates JR Complement factor H genetic variant and age-related macular degeneration: effect size, modifiers and relationship to disease subtype. Int J Epidemiol (2012) 41:250–62 10.1093/ije/dyr204 22253316 PMC3304526

[21] Ambati J Atkinson JP Gelfand BD Immunology of age-related macular degeneration. Nat Rev Immunol (2013) 13:438–51 10.1038/nri3459 23702979 PMC3941009

[22] Prosser BE Johnson S Roversi P Herbert AP Blaum BS Tyrrell J Structural basis for complement factor H-linked age-related macular degeneration. J Exp Med (2007) 204:2277–83 10.1084/jem.20071069 17893204 PMC2118454

[23] Clark SJ Bishop PN Day AJ Complement factor H and age-related macular degeneration: the role of glycosaminoglycan recognition in disease pathology. Biochem Soc Trans (2010) 38:1342–8 10.1042/BST0381342 20863311

[24] Day AJ Clark SJ Bishop PN Understanding the molecular basis of age-related macular degeneration and how the identification of new mechanisms may aid the development of novel therapies. Expert Rev Ophthalmol (2012) 6:123–8 10.1586/eop.11.10

[25] Williamson KA Hamilton A Reynolds JA Sipos P Crocker I Stringer SE Age-related impairment of endothelial progenitor cell migration correlates with structural alterations of heparan sulfate proteoglycans. Aging Cell (2013) 12:139–47 10.1111/acel.12031 23190312

[26] Feyzi E Saldeen T Larsson E Lindahl U Salmivirta M Age-dependent modulation of heparan sulfate structure and function. J Biol Chem (1998) 273:13395–8 10.1074/jbc.273.22.13395 9593669

[27] Lee HY Han L Roughley PJ Grodzinsky AJ Ortiz C Age-related nanostructural and nanomechanical changes of individual human cartilage aggrecan monomers and their glycosaminoglycan side chains. J Struct Biol (2013) 181:264–73 10.1016/j.jsb.2012.12.008 23270863 PMC3578138

